# Few Sex Differences in Hospitalized Suicide Attempters Aged 70 and Above

**DOI:** 10.3390/ijerph15010141

**Published:** 2018-01-16

**Authors:** Stefan Wiktorsson, Therese Rydberg Sterner, Madeleine Mellqvist Fässberg, Ingmar Skoog, Anne Ingeborg Berg, Paul Duberstein, Kimberly Van Orden, Margda Waern

**Affiliations:** 1 Institute of Neuroscience and Physiology, Department of Psychiatry, University of Gothenburg, Blå Stråket 15, SU/Sahlgrenska, 413 45 Gothenburg, Sweden; margda.waern@gu.se; 2 Institute of Neuroscience and Physiology, Department of Psychiatry, University of Gothenburg, Wallinsgatan 6, SU/Sahlgrenska, 431 41 Mölndal, Sweden; therese.rydberg@gu.se (T.R.S.); madeleine.mellqvist@gu.se (M.M.F.); ingmar.skoog@gu.se (I.S.); 3 Institute of Psychology, University of Gothenburg, Haraldsgatan 1, 413 14 Gothenburg, Sweden; anne.berg@psy.gu.se; 4 University of Rochester Medical Center, 300 Crittenden Blvd, Box Psych, Rochester, NY 14642, USA; paul_duberstein@urmc.rochester.edu (P.D.); kimberly_vanorden@urmc.rochester.edu (K.V.O.)

**Keywords:** sex differences, suicide attempt, late life, depression, physical disability

## Abstract

Relatively little research attention has been paid to sex issues in late life suicidal behaviour. The aim was to compare clinical characteristics of women and men aged 70+ who were hospitalized after a suicide attempt. We hypothesized higher depression and anxiety scores in women, and we expected to find that men would more often attribute the attempt to health problems and compromised autonomy. Participants (56 women and 47 men, mean age 80) were interviewed by a psychologist. In addition to psychiatric and somatic health assessments, participants responded to an open-ended question concerning attributions of the attempt. There were no sex differences in depression and anxiety. Forty-five percent of the men and 14% of the women had a history of substance use disorder (*p* = 0.02). At least one serious physical disability was noted in 60.7% of the women and 53.2% of the men (*p* = 0.55). Proportions attributing their attempt to somatic illness did not differ (women, 14.5% vs. men 17.4%, *p* = 0.79), and similar proportions attributed the attempt to reduced autonomy (women, 21.8% vs. men, 26.1%, *p* = 0.64). We found strikingly similar figures for depression scores, functional disability and attributions for attempting suicide in older men and women. Larger studies are needed in diverse settings as sex differences might be influenced by cultural context.

## 1. Introduction

Suicide prevention interventions that target older people appear to be more successful in women than in men [1]. This presents a public health challenge, as suicide rates are particularly high among older men in most countries worldwide. It has been suggested that those who survive suicide attempts in later life constitute a group that could provide keys to the prevention of suicide in later life [2]. It is therefore surprising that relatively little research attention has been paid to sex and gender issues in clinical presentations of older people with suicidal behaviour.

We could identify no study that focused on sex differences in psychopathology in older persons who presented at hospital after a suicide attempt. Regarding suicidal behaviour with fatal outcome, there is some evidence from both mixed age [3] and older adult [4,5] cohorts that symptoms of depression and anxiety are more common in women who die by suicide compared to their male counterparts. This may in part reflect the sex differential in depression rates in underlying populations. While the sex gap tends to decrease with age, many reports suggest a female preponderance of anxiety and depression in later life [6,7,8,9]. A Canadian study identified mental disorders as the major precipitant stressor in older women who died by suicide, but physical illness was the main stressor in men [4]. Findings from our own psychological autopsy study [10] showed that serious physical illness/disability was associated with a four-fold increase in suicide risk in men aged 65 and above, but no association was found in women. Men may be more vulnerable to react with suicidal behaviour when faced with physical illness and disability. The social roles to which men aspire are often thought to require higher levels of physical function and vigor. Loss of vigor/function may be perceived by many men as having adverse consequences for their identity. The authors of a qualitative study on end of life plans in older adults with chronic health issues noted that men emphasized that physical illness threatened their need to be independent and powerful [11]. Suicide was brought up by these men as a means of regaining dignity and control. Paralleling these results, a study of attitudes toward suicide in community living adults [12] revealed that suicide in connection with ill-health was considered more rational, more courageous and more acceptable than suicide under other conditions, especially among men. Men tend to use more lethal suicide methods in connection with suicide, and within a given method greater lethality and greater case fatality is observed in men [13,14]. In a large cross-national study, serious suicide attempts were more prevalent among male attempters [15] compared to females. The late life literature on this topic is sparse. A US-based study compared suicide intent scores in men and women aged 70 and above with recent suicide attempts. Women scored numerically (but non-significantly) higher on the Suicide Intent Scale [16].

The aim of the current study was to examine potential sex differences in clinical presentations of older women and men who were hospitalized after a suicide attempt. Data stem from a larger study on attempted suicide in older adults in western Sweden [17]. Previously unpublished gender-specific data are presented in the current study. Basic sociodemographic and clinical data, including diagnoses, functional disability, history of suicidal behaviour and psychiatric treatment as well as ratings of global cognition, suicide intent and depression-related symptoms are compared in men and women. In light of the findings outlined in the above introduction, we hypothesized that women would exhibit more severe symptoms of depression and anxiety. Further, we anticipated that physical disability would be more common in male attempters, and that men would be more likely to spontaneously attribute their attempt to compromised physical health and functional disability when asked the open-ended question “Why did you attempt suicide?”

## 2. Materials and Methods

### 2.1. Participants

Fifty-six women and 47 men aged 70 and above who were admitted to medical emergency departments in connection with a suicide attempt were included in the study [17]. A suicide attempt was defined as “a situation in which a person has performed an actual or seemingly life-threatening behavior with the intent of jeopardizing his or her life, or to give the appearance of such an intent but which has not resulted in death” [18]. Participants were recruited from 5 hospitals in western Sweden during 3 years (2003–2006). Pre-specified exclusion criteria [17] included terminal illness (*n* = 2), a score below 15 on the Mini Mental State Examination (MMSE) [19] (*n* = 2) and insufficient knowledge of the Swedish language (*n* = 1). Twenty-eight persons declined participation. Seven persons left hospital without study information and two died before the scheduled interview, yielding 103 participants. This corresponds to a participation rate of 77.4% (74.6% for women and 81.0% for men, *p* = 0.384). Mean age did not differ between women and men (mean 80.5, SD, 5.2, vs. 78.7, SD, 5.4, t = −1.641, df = 101, *p* = 0.104).

### 2.2. Procedures

A psychologist (SW) licensed by the Swedish Board of Health and Welfare performed all interviews. The psychologist had 14 years of clinical experience in psychiatric services as well as 2 years of research experience in the field of geriatric psychiatry. The mean time between the suicide attempt and the interview was 15.3 days (for men, 15.5 and for women 15.0 days, *p* = 0.833). The mean duration of inpatient psychiatric treatment was 30.3 days for women (SD = 17.9, range = 1–82 days) and 25.2 days for men (SD = 17.9, range = 1–75 days), *p* = 0.178. The interview protocol was based on the standardized protocol used in the H70 Neuropsychiatric Epidemiology studies, as described in [20]. In addition to this standardized protocol, two well-established clinical rating scales were added for the purpose of this study, the Geriatric Depression Scale and the Suicide Intent Scale (described below).

### 2.3. Major and Minor Depression

An algorithm based on the Comprehensive Psychopathological Rating Scale (CPRS) [21] and in accordance with the DSM-IV [22] was used to diagnose major depression [23]. Minor depression [17] was diagnosed in accordance with DSM-IV research criteria.

### 2.4. Substance Abuse or Dependence

A diagnosis of lifetime alcohol use disorder was based on interview data, medical record review or the regional hospital discharge register and 21 men and 6 women were identified by this procedure [24]. Review of medical records showed that seven women and three men had a history of prescription drug dependence, all but two of whom (both women) also had a lifetime alcohol use disorder.

### 2.5. Dementia

The neuropsychological examination included the MMSE [19] and tests of short- and long-term memory, abstract thinking, aphasia, apraxia and agnosia as described previously [23]. A research diagnosis of dementia was made in accordance with DSM-III-R [25] using the algorithm developed by Skoog et al. [23] which requires: a. Impairment in short-and long-term memory and b. At least one of: b1/ Impairment in abstract thinking, b2/ Aphasia, b3/ Agnosia and b4/ Personality change.

### 2.6. Physical Disability

A physician who was unfamiliar with the aim of the study and blind to participants’ sex applied the World Health Organization description of disability (“an umbrella term, covering impairments, activity limitations, and participation restrictions”) [26] while reviewing organ level data from the Cumulative Illness Rating Scale for Geriatrics (CIRS-G) [27,28]. For the purpose of this study a participant was considered to have a serious disability when at least one impairment, activity limitation or participation restriction reached a level of 3 (severe/constant disability) or 4 (extremely severe disability) on the CIRS-G.

### 2.7. The Comprehensive Psychopathological Rating Scale (CPRS)

The CPRS [21] was used to examine psychiatric symptoms during the month prior to the suicide attempt. This scale comprises 67 psychiatric symptoms, 40 of these are reported and the remaining 27 are observed and rated by the interviewer. The CPRS has been shown to be applicable and reliable in older adult clinical cohorts, influenced by neither aging nor mild cognitive impairment, demonstrating reliability data on par with that of younger populations [29]. Each CPRS item is scored from 0 to 6, with 6 indicating the most severe level of symptoms and 0 the absence of the symptom. For the purpose of this study, we examined 18 of these symptoms. A symptom was defined as present when the score was 2 or more, as a score of 1 was considered to be within the range of normal psychopathology. The Montgomery-Åsberg Depression Rating Scale (MADRS) [30], derived from the CPRS, was used to rate depressive symptoms during the month preceding the index attempt. This scale includes ten items, yielding a maximum score of 60. The Brief Scale for Anxiety (BSA) [31], also derived from the CPRS, provides an overall rating of anxiety burden. We used a modified 9-item version (phobia question excluded), with a maximum score of 54.

### 2.8. Geriatric Depression Scale (GDS)

We also employed a depression scale developed specifically for older adults, the Geriatric Depression Scale (GDS), which has been validated for this age group [32]. The 20 item Swedish version was used in the current study [33]. As the phenomenon of hopelessness has central relevance in the study of suicidal behavior, and the MADRS includes no measure of hopelessness, we the hopelessness item (“Do you think your situation is hopeless?”) was utilized as a single item.

### 2.9. Suicide Intent Scale (SIS)

Suicide intent was measured using the Suicide Intent Scale [34]. This scale comprises 8 objective and 7 subjective items. Items are scored from 0 (low intent) to 2 (high intent) yielding a maximum score of 30. The scale has been shown to have predictive validity regarding future suicide in older adult suicide attempters [35].

### 2.10. Reasons for Attempting Suicide

An open-ended question, “Why did you attempt suicide?” was used to explore attributions for the attempt. The participant’s spontaneous response was recorded verbatim. There were no flash cards, and no follow-up questions. The details of the coding process and qualitative content analysis are described in [36]. Nine attributions were identified: somatic problems and pain, functioning and autonomy, psychological problems, social problems, lack of meaning, perceived burden and escape, no memory or understanding and wanted to die without a specific reason.

### 2.11. Ethics

The Research Ethics Committee at the University of Gothenburg approved the study (S 063-03, approval date 2003-02-18). All the participants provided written consent following oral and written information about the study.

### 2.12. Statistics

Due to small numbers, Fisher’s exact test was employed to test for sex differences in proportions. The Mann-Whitney U test was applied to test for sex differences regarding number of depressive, anxiety, cognitive and somatic symptoms related to depression and data were further tested in an ordinal regression model. Assumptions of ordinal regression were fulfilled. The *t*-test was used to test for potential sex differences in means. As age and education level strongly influence MMSE score, a linear regression including these variables was performed. As SIS scores did not show a normal distribution, the Mann-Whitney U test was applied to test for sex differences. All statistical tests were two-sided. Statistical significance was determined where *p*-values were less than 0.05. The Bonferroni correction method was applied to address the problem of multiple comparisons.

## 3. Results

Sociodemographic and diagnostic characteristics of the sample are presented in Table 1. Two thirds of the women fulfilled criteria for major depression, a proportion similar to that observed in men. Rates of minor depression were also similar. Forty-five percent of the men had a history of alcohol/substance use disorder, a proportion significantly larger than that in women also after the Bonferroni correction. Over two thirds of the women reported that they found their situation hopeless, a proportion that was significantly higher than that for men (43.8%), but the difference was not statistically significant adjustment for multiple comparisons. Proportions using violent methods were nearly identical in women and men. Six out of ten of the women and slightly more than half of the men had at least one serious physical disability.

Table 2 shows proportions scoring 2 or above on CPRS depression-related symptoms. None of the specific symptoms were related to sex, nor were the number of symptoms in specified categories. Results of the ordinal regression are shown in Table S1. MADRS scores were almost identical in women and men and similar depression scores were observed on the GDS (Table 3). Global cognitive capacity, as measured with MMSE, was higher in men. The sex difference regarding MMSE remained in the linear regression model that included age and education level (Beta = −1.764, SE = 0.708, *p* = 0.015). However, it did not survive the Bonferroni correction. No sex differences were observed regarding suicide intent. Identical medians were observed in men and women for the SIS total score, as well as the subjective subscale.

**Figure 1:**
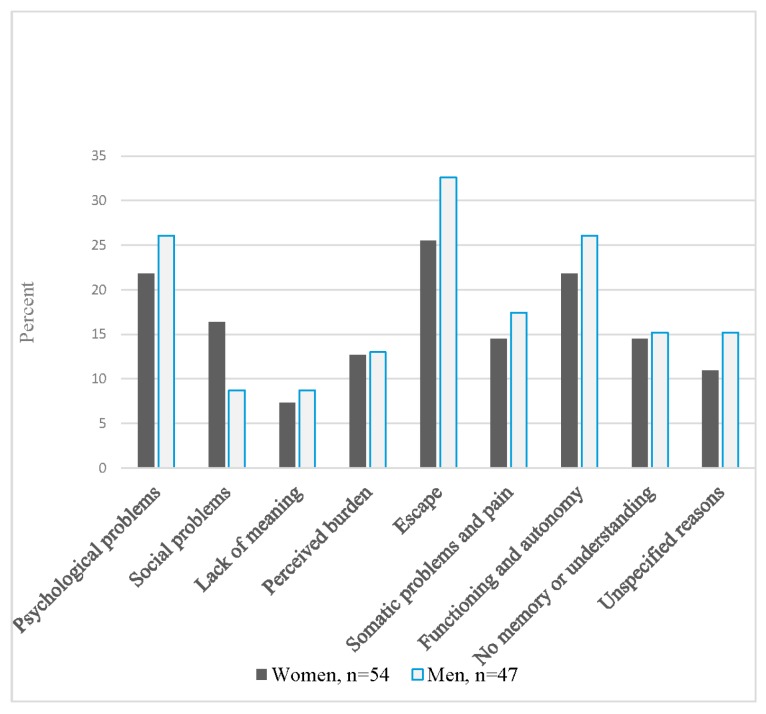
Self-reported reasons ^a^ for attempting suicide among hospitalized suicide attempters ^b^ aged 70 and above, by sex. ^a^ Participants could report more than one reason; ^b^ Missing data for two women.

Self-reported attributions for the attempt are shown in Figure 1. Proportions reporting that the attempt was due to somatic problems and pain did not differ between women and men. Similar proportions were also seen regarding functioning and autonomy. Results of significance testing for comparison of attributions in women and men are shown in Table S2.

## 4. Discussion

Contrary to our hypotheses, depression scores, functional disability and self-reported reasons for attempting suicide were strikingly similar in women and men in this northern European cohort of older adults hospitalized in connection with a suicide attempt. An important consideration is that our study may have been underpowered to detect some potential sex differences. Another partial explanation for the lack of sex differences in our study might be related to survival bias, as the chance of surviving suicidal acts is smaller in men than in women. Women and men who survive suicidal behaviour may share common clinical characteristics and attributions to a greater degree than those who die. Another explanation might be related to the high age of our cohort, as sex differences may decrease with age [38].

Only one clinical variable survived the Bonferroni adjustment. A lifetime history of substance abuse/dependence was noted in half of the men. The proportion was identical to that reported for participants aged 70 and above in the above cited U.S. study [16]. Proportions were lower for women in both studies, which may reflect lower rates of substance misuse in the background population [24].

Two different ratings scales were employed to rate affective psychopathology and both showed highly comparable ratings in women men and, less than a single point for both measures, indicating a lack of clinical significance as well as statistical significance.

Sixty-one percent of the women and 53% of the men had serious physical disabilities. We recently reviewed the literature on physical illness, functional disability and suicidal behavior in older adults [39], and found no clinical reports regarding physical disabilities in older women and men with suicide attempts for comparison. Our findings expand on those of a population-based European multicenter study that found an association between functional disability and death wishes, with no sex difference [40].

Proportions attributing the attempt to physical problems and pain did not differ by sex and we found no differences regarding the attributions functioning and autonomy. While we could find no similar study for comparison, we do note that a U.S. study involving community-dwelling older adults [41] found that greater value placed on autonomy among men amplified the relation between depressive symptoms and suicide risk. This was not the case for women in that study.

The women in our study scored slightly lower on the MMSE compared to men, but the association did not survive the Bonferroni correction. In general, research on cognitive aging suggests that women tend to outperform men on many cognitive tasks in late life. However, a recent Japanese study [42] on community-dwelling older adults found a modest effect of sex with lower MMSE scores among women in the oldest age group. The sex difference in MMSE scores in our study is unlikely to be fully explained by differences in education, age, major depression or the number of days between the index attempt and the research interview. It is possible that women’s suicide attempts were more likely to have specific cognitive or neurologic sequelae, which could have led to their slightly (non-significantly) lower MMSE scores. Our methods were however unable to detect such sequelae.

In the current study we found similar ratings of suicide intent in men and women as measured by the SIS. This expands on our previously reported finding of a lack of sex difference in medical severity in the same cohort [43]. We note that, despite some methodological differences, intent scores in the current study were very similar to those reported for the 70+ age group in the U.S. study that focused on patients with major depression [16]. The number of participants in the 70+ group in that study was limited and results regarding sex difference were inconclusive. Although the current study is larger, it was not powered to detect relatively small differences in SIS scores. Larger cohorts will be needed to further address the question of sex differences in suicide intent among older adults with non-fatal suicidal behavior.

## 5. Methodological Considerations

One strength of our study is the high age cut-off for inclusion. The interviewer was a male clinician with extensive experience in conducting research interviews with older adults, which might have helped both men and women to feel more comfortable when talking about their symptoms in a research interview. While this is one of the largest clinical samples of older individuals who attempt suicide, the study was not powered to detect relatively small effect sizes. In other words, there may be sex differences in this population, but our sample size was too small to detect these differences. Lack of statistical power can lead to conclusions that are not reliable or robust. Another limitation is that the data were collected more than 10 years ago. Data were collected by a single psychologist, and beyond the use of a structured protocol, no attempts were made to account for or minimize bias. Our study lacked a measure of masculinity and femininity, which are important to consider as masculinity, rather than biological sex, was found to be associated with serious suicidal thoughts in older, but not younger cohorts [44]. Participants can be considered representative of the underlying population in terms of sociodemographics [17]. However, it must be stressed that our study design can capture only those who are in contact with hospital services after a suicide attempt.

Our findings cannot be extrapolated directly to other cultural settings. Normative and social structures of femininity and masculinity can differ between cultures and change over time [45,46] as do suicide rates [47]. In 2016 suicide rates were at a moderately high level in Sweden, 13.2/100,000 in women and 28.6/100,000 for men aged 70 and above. Corresponding figures for non-fatal suicidal behavior were 51.7/100,000 in women and 52.8/100,000 in men. One important issue is that shooting is a relatively uncommon method in Sweden as it is in the rest of Europe. This may influence patterns of both fatal and non-fatal behaviors. Over three-quarters of the participants in our study were hospitalized in connection with an overdose [43], a larger proportion than that (63%) reported in a U.S. study [48].

## 6. Implications

The study can provide some leads for future research. Our finding regarding affective psychopathology should be seen in light of the relatively high prevalence of antidepressant use in both sexes in our study, which would be expected to impact on symptom presentation. Studies based in other settings are needed, as prescription rates, as well as possible sex differentials in prescription patterns [49] may vary across countries. Future research will also be required to address measures of gender role orientation and attitudes, and these studies will need to include also depressed older women and men without suicide attempt history for comparison.

While the finding of a vast overrepresentation of substance misuse among male suicide attempters is not new, it highlights the pressing need for interventions for suicidal older adult men with substance use issues. Effective treatments are available for older adults with substance misuse. In fact, there is some evidence that older persons have better adherence to both medication and therapy compared to their younger counterparts [50].

Suicidal older adults with functional disabilities comprise another group that needs to be targeted in prevention research. Our finding that functional disability was present in over half of the men and women in this study, taken together with the finding that autonomy was one of the most important attributions for attempting suicide in sexes highlights the need for intervention research to develop strategies to strengthen the older adult’s ability to deal with everyday life issues. Person-centered interventions, based on the suicidal person’s own narrative could be tested. Such interventions could aim at strengthening the older adult’s feeling of capability [51] by facilitating opportunities for the person to do and be what they value, even in the face of significant health issues.

## 7. Conclusions

With the exception of the predominance of substance use issues in men, we found few sex differences in this northern European cohort of suicide attempters aged 70 and above. Larger studies are needed in varied cultural settings, to further inform the development of appropriate interventions.

## Figures and Tables

**Table 1:** Sociodemographic and clinical characteristics of hospitalized suicide attempters aged 70 and above, by sex.

Characteristics	Women *n* = 56		Men *n* = 47		Test Results ^a,b^
	*n*	(%)	*n*	(%)	*p*-Value
**Sociodemographics**					
Married/cohabiting	15	(26.8)	18	(38.3)	0.289
Living alone	41	(73.2)	29	(61.7)	0.289
Living in an institution	4	(7.1)	2	(4.3)	0.686
Education, only mandatory	31	(55.4)	27	(57.4)	0.845
**Clinical Characteristics**					
Major depression	38	(67.9)	30	(63.8)	0.682
Minor depression	13	(23.2)	14	(29.8)	0.504
Alcohol/substance use disorder ^c^	8	(14.3)	21	(44.7)	0.001
Dementia	4	(7.1)	4	(8.5)	1.000
Hopelessness	36	(67.9)	19	(43.8)	0.023
Psychiatric treatment ^c^	34	(60.7)	27	(57.4)	0.841
Current antidepressant prescription	36	(64.3)	26	(55.3)	0.421
Previous suicide attempt	23	(41.1)	14	(29.8)	0.303
Violent method at index attempt ^d^	16	(28.6)	13	(27.7)	1.000
Any serious physical disability ^e^	34	(60.7)	25	(53.2)	0.549

^a^ Fisher’s exact test; ^b^ Bonferroni-adjusted significance threshold, *p* = 0.0036; ^c^ Lifetime history; ^d^ Hanging, cutting, drowning, and other violent methods [37]; ^e^ At least one disability with a score of 3 or 4 in any physical organ category in accordance with the CIRS-G.

**Table 2:** Psychiatric symptoms in accordance with the Comprehensive Psychopathological Rating Scale (CPRS) ^a^ in hospitalized suicide attempters aged 70 and above by sex.

CPRS-Symptoms	Women *n* = 56		Men *n* = 47		Test Results ^b^
	*n*	(%)	*n*	(%)	*p*-Value
**Depressive Symptoms**					
Sadness (reported)	47	(83.9)	43	(93.5)	0.217
Sadness (observed)	49	(87.5)	41	(87.2)	1.000
Inability to feel	44	(80.0)	37	(80.4)	1.000
Pessimistic thoughts	33	(60.0)	30	(63.8)	0.838
Hostile feelings	11	(20.4)	10	(22.4)	1.000
Suicidal thoughts	49	(89.1)	41	(89.1)	1.000
**Anxiety Symptoms**					
Inner tension	28	(51.9)	18	(40.0)	0.312
Worrying over trifles	32	(59.3)	22	(48.9)	0.319
Autonomic disturbances	12	(22.2)	5	(11.1)	0.185
Muscular tension (reported)	27	(49.1)	14	(31.1)	0.102
**Cognitive Symptoms**					
Failing memory	36	(65.5)	26	(57.8)	0.535
Fatiguability	43	(78.2)	32	(71.1)	0.489
Indecision	25	(45.5)	14	(31.1)	0.156
Lassitude	41	(75.9)	32	(71.1)	0.650
Concentration difficulties	27	(50.0)	25	(55.6)	0.687
**Somatic Symptoms**					
Aches and pain	29	(53.7)	23	(51.1)	0.842
Reduced sleep	22	(40.7)	17	(37.8)	0.838
Reduced appetite	26	(47.3)	21	(45.7)	1.000
**Number of Symptoms by Group**					
	Women	Men		Test Results ^c^	
	Median	Median		Z-value	*p*-value
Depressive symptoms	5	5		−0.585	0.559
Anxiety symptoms	2	1		−1.673	0.094
Cognitive symptoms	1	1		−1.009	0.313
Somatic symptoms	0	0		−0.375	0.708

^a^ A symptom was considered to be present when the score was 2 or more; ^b^ Fisher’s exact test; ^c^ Mann-Whitney U Test.

**Table 3:** Results of analyses for rating scales in hospitalized suicide attempters aged 70 and above, by sex.

Rating Scales	Women *n* = 56		Men *n* = 47		Test Results ^a,b^			
	Mean SD		Mean SD		t	df	*p*-Value	Cohen’s d
Montgomery-Asberg Depression Rating Scale (MADRS)	26.6	11.3	26.4	11.4	−0.08	95	0.939	0.02
Geriatric Depression Scale (GDS)	10.1	4.5	9.3	5.0	−0.76	95	0.448	0.17
Brief Scale for Anxiety (BSA)	10.2	6.1	8.4	5.0	−1.56	96	0.122	0.32
Mini Mental State Examination (MMSE)	24.8	3.8	26.5	2.7	2.48	95	0.015	−0.52
**Suicide Intent Scale (SIS)**								
	Women		Men		Test Results ^c^			
	Median		Median		Z-value		*p*-value	
SIS total score	17		17		−0.521		0.602	
SIS objective score	5.5		6		−0.585		0.559	
SIS subjective score	11		11		−0.167		0.867	

^a^ *t*-test, equal variances assumed; ^b^ The Bonferroni-adjusted significance threshold, *p* = 0.0125; ^c^ Mann-Whitney U Test.

## Supplementary Materials

The following are available online at www.mdpi.com/1660-4601/15/1/141/s1, Table S1: Ordinal regression models estimating associations between number of symptoms in specified group and sex, Table S2: Self-reported reasons for attempting suicide among female (*n* = 56) and male (*n* = 47) hospitalized suicide attempters aged 70 and above.
