# Supplementary material for: Few Sex Differences in Hospitalized Suicide Attempters Aged 70 and Above
Source: Int J Environ Res Public Health. 2018 Jan 16;15(1):141. doi: 10.3390/ijerph15010141 (PMC5800240; doi:10.3390/ijerph15010141)
Supplement: Supplementary file 1 [file ijerph-15-00141-s001.pdf]

Supplementary material:

Table 1. Ordinal regression models estimating associations between number of symptoms in specified group and sex.

Symptoms	Estimate	Standard Error (SE)	t-Value	*p*-Value	Odds Ratio (OR)
Depression (ranked: 0–6)					
Sex Women (Baseline group: Men)	−0.218	0.371	−0.589	0.556	0.804
Anxiety (ranked: 0–4)					
Sex Women (Baseline group: Men)	0.608	0.363	1.672	0.095	1.836
Cognitive (ranked: 0–5)					
Sex Women (Baseline group: Men)	−0.382	0.373	−1.024	0.306	0.683
Somatic (ranked: 0–2)					
Sex Women (Baseline group: Men)	0.165	0.437	0.377	0.706	1.179

Table 2. Self-reported reasons for attempting suicide[a] among female (n = 56) and male (n = 47) hospitalized suicide attempters aged 70 and above.

	Women [b] n = 56		Men n = 47		Test Results [c]
	n	(%)	n	(%)	p-Value
Psychological problems	12	(21.8)	12	(26.1)	0.645
Social problems	9	(16.4)	4	(8.7)	0.372
Lack of meaning	4	(7.3)	4	(8.7)	1.000
Perceived burden	7	(12.7)	6	(13.0)	1.000
Escape	14	(25.5)	15	(32.6)	0.510
Somatic problem sand pain	8	(14.5)	8	(17.4)	0.787
Functioning and autonomy	12	(21.8)	12	(26.1)	0.645
No memory or understanding	8	(14.5)	7	(15.2)	1.000
Wanted to die without a specific reason	6	(10.9)	7	(15.2)	0.563

[a] Subjects could report several reasons; [b] Data missing for 2 women; [c] Fisher's exact test.
